# Conservative dentistry and endodontics in India

**DOI:** 10.4103/0972-0707.57629

**Published:** 2009

**Authors:** S Mahalakshmi

**Affiliations:** Department of Conservative Dentistry, SRM Dental College & Hospitals, Ramapuram, Chennai E-mail: dr.mahalaxmis@gmail.com

As my tenure as the President of the FODI is coming to an end, it gives me immense pleasure in communicating to you all through the official journal of the FODI. At the forefront, I would like to congratulate and acknowledge the immense work done by the Editor-in-Chief Dr. Gopi Krishna in bringing out a world class journal for the specialty of Conservative Dentistry and Endodontics in India.

While Endodontics has gained huge momentum, in recent years Conservative Dentistry seems to have been delegated to a backseat in our academic curriculum. While postgraduates are doing more number of molar endodontic cases, the number of conservative cases is diminishing. At the same time, more and more studies are being done in Endodontics while there seems to be less interest in taking up studies in Conservative and Restorative Dentistry.

We are, generally speaking, greatly lacking in research activities and publications. Most of the research activities are done for the sake of completion of the MDS curriculum. If we take efforts to do proper studies and publish these studies and the dissertations done by the postgraduate students, we will have a minimum of two publications per year. Except for a few postgraduate institutions who publish regularly, the rest of us do not have much publications to our credit.

Another worrying trend is the lukewarm response to the scientific sessions at various national and regional conferences and CDE programs among our fellow colleagues and postgraduate students. Such programs have become a social gathering of sorts for the participants. There is more number of participants outside rather than inside the halls! Now that CE credits have become mandatory, the attendance to scientific sessions should be made compulsory for all and some sort of a system has to be adopted to ensure that the participant has really earned the CE points!

The phenomenal efforts taken by our President of the Dental Council, Brigadier Dr. Anil Kohli in the form of CE points for the renewal of registration, publications for academic promotions, etc. are milestones in encouraging more and more research activities and publications incorporating many steps in the academic progress to streamline and put Indian dentistry on the global arena.

Following his footsteps, under the banner of FODI, SPIRIT 2009 was conducted by the Department of Conservative Dentistry and Endodontics, SRM Dental College, Chennai. “Spirit” by definition refers to an attitude or principle that inspires, animates, or pervades to thoughts, feelings, or action. SPIRIT by our definition is the science based on principles, inventions, research, and innovative thoughts.

The event was inaugurated by Dr. Anil Kohli who appreciated the different innovative models on display. Postgraduate students and faculty members from various institutions across the country put their brains together to bring out mind-boggling scientific models. As a highlight of this event, we had organized two “video conferences,” one from Canada by Dr. Glenn Van As and the other from New Zealand by Dr. CVN Rao. Seeing the enthusiastic participation of the delegates, I am sure that such events can be conducted regularly on an annual basis during our national conferences and postgraduate conventions along with the paper/poster presentations.

I once again sincerely wish to thank Dr. Anil Kohli, President, DCI, Dr. Lakshmi Narayanan, Secretary, FODI, and all the members for having faith in me and entrusting this post. I bid adieu at this juncture, but will continue to work for the benefit of the organization for years to come.



